# Enhanced anti-tumor effects by combination of tucatinib and radiation in HER2-overexpressing human cancer cell lines

**DOI:** 10.1186/s12935-024-03458-3

**Published:** 2024-08-06

**Authors:** Lukas Amrell, Eric Bär, Annegret Glasow, Rolf-Dieter Kortmann, Clemens Seidel, Ina Patties

**Affiliations:** 1 Department of Radiation Oncology, University of Leipzig, Stephanstraße 9a, 04103 Leipzig, Germany; 2 Comprehensive Cancer Center Central Germany (CCCG), Leipzig, Germany

**Keywords:** HER2, Tucatinib, Radiotherapy, Breast cancer, Colorectal cancer, Non-small cell lung cancer

## Abstract

**Background:**

Tucatinib (TUC), a HER2-directed tyrosine kinase inhibitor, is the first targeted drug demonstrating intracranial efficacy and significantly prolonged survival in metastatic HER2-positive breast cancer (BC) patients with brain metastases. Current treatments for brain metastases often include radiotherapy, but little is known about the effects of combination treatment with TUC. Therefore, we examined the combined effects of irradiation and TUC in human HER2-overexpressing BC, non-small cell lung cancer (NSCLC), and colorectal cancer (CRC) cell lines. For the latter two, a standard therapy successfully targeting HER2 is yet to be established.

**Methods:**

Nine HER2-overexpressing (BC: BT474, ZR7530, HCC1954; CRC: LS411N, DLD1, COLO201; NSCLC: DV90, NCI-H1781) and three control cell lines (BC: MCF7, HCC38; NSCLC: NCI-H2030) were examined. WST-1 assay (metabolic activity), BrdU ELISA (proliferation), γH2AX assay (DNA double-strand breaks (DSB), Annexin V assay (apoptosis), and clonogenic assay (clonogenicity) were performed after treatment with TUC and/or irradiation (IR). The relevance of the treatment sequence was analyzed exemplarily.

**Results:**

In BC, combinatorial treatment with TUC and IR significantly decreased metabolic activity, cell proliferation, clonogenicity and enhanced apoptotis compared to IR alone, whereby cell line-specific differences occurred. In the *PI3KCA*-mutated HCC1954 cell line, addition of alpelisib (ALP) further decreased clonogenicity. TUC delayed the repair of IR-induced DNA damage but did not induce DSB itself. Investigation of treatment sequence indicated a benefit of IR before TUC versus IR after TUC. Also in CRC and NSCLC, the combination led to a stronger inhibition of metabolic activity, proliferation, and clonogenic survival (only in NSCLC) than IR alone, whereby about 10-fold higher concentrations of TUC had to be applied than in BC to induce significant changes.

**Conclusion:**

Our data indicate that combination of TUC and IR could be more effective than single treatment strategies for BC. Thereby, treatment sequence seems to be an important factor. The lower sensitivity to TUC in NSCLC and particularly in CRC (compared to BC) implicates, that tumor promotion there might be less HER2-related. Combination with inhibitors of other driver mutations may aid in overcoming partial TUC resistance. These findings are of high relevance to improve long-time prognosis especially in brain-metastasized situations given the intracranial activity of TUC.

**Supplementary Information:**

The online version contains supplementary material available at 10.1186/s12935-024-03458-3.

## Background

Breast cancer (BC) is the most common cancer in females and one of the main causes of cancer death worldwide [1]. Several molecular subtypes with different therapeutic options and prognoses are defined based on the expression of estrogen or progesterone receptors and epidermal growth factor receptor 2 (ERBB2/HER2). Approximately 20% of BCs are classified as HER2-positive, indicating overexpression on the cell surface or amplification of the receptor gene [2]. Although HER2 itself has no known direct ligand, it can form potent dimers with other receptors of the same family [3], which then transfer signals through a variety of intracellular cascades like the mitogen-activated protein kinase (MAPK), protein kinase C (PKC), and phosphatidylinositol 3-kinase (PIK3) pathways [4]. The HER2-overexpressing BC subtype is associated with a worse prognosis [5]. With available HER2-targeting therapies, the 5-year relative survival rate increased to about 85% [6]. During the last 20 years, several HER2-targeted therapies, starting with the humanized antibody trastuzumab, had been emerging and have been shown to significantly improve outcome [7, 8]. Nevertheless, despite current therapies, up to 50% of patients with HER2-positive, metastatic BC are reported to develop brain metastases with high rates of intracranial progression [9, 10].

Tucatinib (TUC) is an orally available, reversible HER2-targeted small-molecule tyrosine kinase inhibitor (TKI) approved by the European Medicines Agency (EMA) for the treatment of HER2-positive BC after two HER2-directing therapies. Most importantly, it has shown intracranial efficacy with prolonged overall survival in patients with HER2-positive, metastatic BC in the randomized, placebo-controlled HER2CLIMB trial [11, 12]. Its manageable adverse effect profile and higher selectivity for HER2 compared to other HER2 TKI, such as lapatinib and neratinib [13], makes it an excellent candidate for combination with other therapies. Currently, little is known about the combination of TUC with radiotherapy in cancer patients [14], although stereotactic radiotherapy for limited or whole-brain radiotherapy for extensive brain metastases is part of the standard-of-care (SOC) used for treating brain metastases [15, 16]. Other HER2-targeted therapies have shown that other HER2-directed drugs, such as lapatinib, are able to radiosensitize BC cells [17, 18].

While HER2 is an established subtype marker in BC, the receptor is also overexpressed in a variety of other cancer entities [19] including colorectal cancer (CRC) and non-small cell lung cancer (NSCLC). Reported percentages of HER2-positive tumors are 5.2%, based on HERACLES criteria, for CRC [20] and about 20% in NSCLC [21–23]. As there is no gold standard for HER2 positivity, the data vary greatly depending on the criteria used, including HER2 overexpression and gene amplification [24–26], [23, 27]. In 2023, based on the results of the phase II MOUNTAINEER trial, TUC combined with trastuzumab was approved by the US Food & Drug Administration (FDA) as the first anti-HER2 treatment for metastatic CRC [28]. However, in NSCLC, HER2-targeting therapies are not SOC until today, representing an unmet medical need [29]. Similar to BC, radiotherapy is also applied to CRC and NSCLC at various stages of the disease, e.g., for locally recurrent tumors, oligometastatic disease, or in palliative situations.

In this study, we analyzed for the first time the in vitro effects of a combinatorial treatment with TUC and irradiation (IR) in HER2-overexpressing BC, CRC, and NSCLC cell lines.

## Methods

### Cell lines and cell cultures

Human BC cell lines BT474, HCC1954, HCC38 and MCF7 were kindly provided by PD Dr. Claudia Stäubert of the Rudolf Schönheimer Institute of Biochemistry, University of Leipzig, Germany. ZR7530 was purchased from the European Collection of Authenticated Cell Cultures (ECACC, Salisbury, UK). CRC cell lines COLO201 and DLD1 were also provided by PD Dr. C. Stäubert, while LS411N was purchased from the American Type Culture Collection (ATCC, Manassas, VA, USA). NSCLC cell lines DV90, NCI-H1781, and NCI-H2030 were purchased from ATCC, and A549 was purchased from the German Collection of Microorganisms and Cell Cultures (DSZM, Braunschweig, Germany). BT474, HCC1954, HCC38, MCF7, COLO201, DLD1, LS411N, NCI-H1781, and NCI-H2030 were cultured in RPMI 1640 (Gibco, Thermo Fisher Scientific, Waltham, MA, USA), ZR7530 in RPMI 1640 supplemented with 10 mmol/l HEPES (Lonza, Basel, Switzerland), DV90 in RPMI 1640 supplemented with 1% MEM non-essential amino acids (Gibco, Thermo Fisher Scientific, Waltham, MA, USA), and A549 in DMEM (Lonza, Basel, Switzerland). All media were supplemented with 10% fetal calf serum (FCS; Sigma‒Aldrich, St. Louis, MA, USA), 100 U/ml penicillin and 100 µg/ml streptomycin (Lonza, Basel, Switzerland). To RPMI 1640, also 2.5 mg/ml D(+)-glucose (AppliChem, Darmstadt, Germany) and 110 µg/ml sodium pyruvate (AppliChem, Darmstadt, Germany) were added. Cell cultures were maintained at 37 °C with 5% CO_2_. Cells were passaged with trypsin/EDTA (Lonza, Basel, Switzerland). All cell lines except for NCI-H2030, MCF7 and HCC38 (controls) were described to overexpress HER2 ( [30] (BC, CRC, NSCLC); [31] (BC); [32] (BC); [33] (CRC); [34] (CRC); [35] (NSCLC); [36] (NSCLC); [37, 38] (NCI-H2030)).

### Irradiation

Cells were irradiated using a 200 kV X-ray machine (Xstrahl 200, Xstrahl, Ratingen, Germany) at dose rates between 1.27 and 1.91 Gy/min, depending on whether irradiation (IR) was performed on cells in well plates, Petri dishes, or chamber slides. IR was applied single time or fractionated (daily, d1-4) as indicated in Table 1.

### Drugs

Tucatinib (TUC, trade name TUKYSA^®^) was kindly provided by Seagen Inc. (Seattle, WA, USA). PI3KCA inhibitor alpelisib (ALP/BYL-719) was purchased from Selleckchem (Houston, TX, USA). Stock solutions of 100 mM TUC and ALP were prepared in dimethyl sulfoxide (DMSO, Sigma‒Aldrich, St. Louis, MA, USA) and stored at -80 °C for long-term or at -20 °C for a maximum of 4 weeks. Working solutions were prepared in culture medium immediately before use.

### Treatment schedule

After seeding (d0), cells were allowed to attach for 24 h. Number of seeded cells in WST-1, BrdU, Annexin-V, and γH2AX assays was chosen so that cells reached a confluence of less than 100% on day of examination. Preliminary experiments have shown no significant effects of single-dose IR (8 Gy) on BCs, in contrast to NSCLC and CRC cells. Therefore, fractionated IR was used in all BC cell experiments. Unless otherwise noted, treatment with TUC was immediately followed by irradiation. The cell treatment schedule was adapted to the cancer entity and assay type, as detailed in Table 1.

**Table 1:** Tucatinib and irradiation treatment schedule

		Tucatinib		Irradiation	
		Single time	Fractionated (daily)	Single time	Fractionated (daily)
BC	Metabolic activity	d1 d4 in BT474	-	-	d1-4
	Proliferation	d1	-	-	d1-4
	Apoptosis	d1	-	-	d1-4
	Clonogenic survival	-	d1-4	-	d1-4
	DSB	d1	-	d1	-
NSCLC and CRC	Metabolic activity	d1	-	d1	-
	Proliferation	d1	-	d1	-
	Apoptosis	d1	-	d1	-
	Clonogenic survival	-	d1-4	-	d1-4

### Metabolic activity

To evaluate treatment effects on metabolic activity, WST-1 assay was performed (Roche, Basel, Switzerland). Cells were seeded in 96-well or 48-well plates. 72 h after last treatment, cell culture medium was discarded, WST-1 reagent (1:10 in culture medium) was added, and absorbance was measured at 435 nm (SpectraMax^®^ i3x, Molecular Devices, San Jose, CA, USA) according to manufacturer’s instructions.

### Proliferation

Cell proliferation was measured by colorimetric 5-bromo-2’-deoxyuridine (BrdU) cell proliferation ELISA (Roche, Basel, Switzerland) according to manufacturer’s instructions. Cells were seeded in 96- or 48-well plates. BrdU was added 4 to 72 h before measurement, depending on the growth rate of the cells. The absorbance at 370 nm was measured with a SpectraMax^®^ i3x (Molecular Devices, San Jose, CA, USA) at 72 h after treatment/the last fraction.

### Apoptosis

To detect apoptosis 72 h after treatment, Annexin-V-FLUOS Staining Kit (Roche, Basel, Switzerland) was used according to manufacturer’s instructions. Alternatively, in BC cell lines and NSCLC cell line A549, FITC-labeled Annexin V (ImmunoTools, Friesoythe, Germany; 1:10 − 1:50 in phosphate-buffered saline with 1 mM CaCl2 and 6 mM MgCl2) was applied. Trypsinized cells were incubated with Annexin V at room temperature for 15–30 min. Propidium iodide (PI) solution (Sigma‒Aldrich, St. Louis, MA, USA; 3 µg/ml) was added immediately before measurement by flow cytometry (BD Accuri C6 Plus, BD Biosciences, Heidelberg, Germany). Fluorescence was examined on at least 5000 cells per sample. Cells were classified as vital (FITC-/PI-), early (FITC+/PI-) or late apoptotic (FITC+/PI+), or necrotic (FITC-/PI+) cells. Early and late apoptotic cells are presented cumulatively as apoptotic cells.

### Clonogenic survival

Clonogenic assay was performed and analyzed as described in [39], with the following changes: cells were seeded in 6-well plates at three different densities in duplicates or in Petri dishes (⌀ 6 cm) at two different densities (HCC1954 only), and treatment started 24 h later. Nine to 28 d after initial plating, colonies were stained as described previously [39].

### DNA double-strand breaks

To evaluate the number of DNA double-strand breaks (DSBs) after treatment of BT474, ZR7530 and HCC1954, γH2AX assay was adapted from [39]. Cells were seeded on 8-well chamber slides (Chamber Slide System 177,445, Nalgene Nunc International, Rochester, NY, USA) and allowed to attach for 24 h. Cells were fixed 1, 24, 48, or 72 h after treatment. Mouse anti-phospho-histone H2A.X (Ser139; clone JBW301, Merck Millipore, Burlington, MA, USA) and Alexa Fluor^TM^ 568 goat anti-mouse (Invitrogen, Waltham, MA, USA) antibodies were used for γH2AX detection. Nuclei were stained with 1 µg/ml DAPI (Sigma‒Aldrich, St. Louis, MO, USA). Statistical analysis was conducted using one-tailed Student’s *t*-test for two independent samples with equal variance on at least 50 nuclei per group.

### HER2 staining

Cells were detached by PBS washing and fixed with 2% formaldehyde for 15 min, permeabilized with 0.5% Triton X-100 and washed (wash buffer: PBS, 0.5% BSA, 200 mM EDTA). After blocking with normal goat serum (NGS, 2% in PBS), a mouse monoclonal antibody (anti-ErbB2 (HER-2) Monoclonal Antibody (e2-4001), Invitrogen™) or corresponding mouse IgG1 negative control (clone Ci4, MABC002, Merck) was applied (0.4 µg/ml in 2% NGS) for 50 min. Cells were washed twice and incubated with F(ab’)2-goat anti-mouse IgG (H + L) cross-adsorbed secondary antibody (Alexa Fluor™ 488 ;1:200; Invitrogen #A-11,017) for 40 min and washed twice. After resuspension in 400 µl wash buffer, a part of cells was analyzed by FACS (BD Accuri C6) and the other part was counterstained with 1 µg/ml DAPI (Sigma) and photographed in 8-well chamber slides (40-fold magnification) using a fluorescence microscope (Keyence BZ-9000, haze reduction 30%).

### Statistics

Statistical analyses were performed with Microsoft Excel 2016 software by using two-tailed Student’s *t*-tests for two samples with equal variance if not otherwise noted. *P* values were considered significant at *p* ≤ 0.05 and are marked with asterisks or hashs for *p* ≤ 0.05 (*/#), *p* ≤ 0.01 (**/##) and *p* ≤ 0.001 (***/###). The data are presented as the mean ± standard error of the mean (SEM), with n representing the number of independent experiments unless otherwise noted.

## Results

**Fig. 1:**
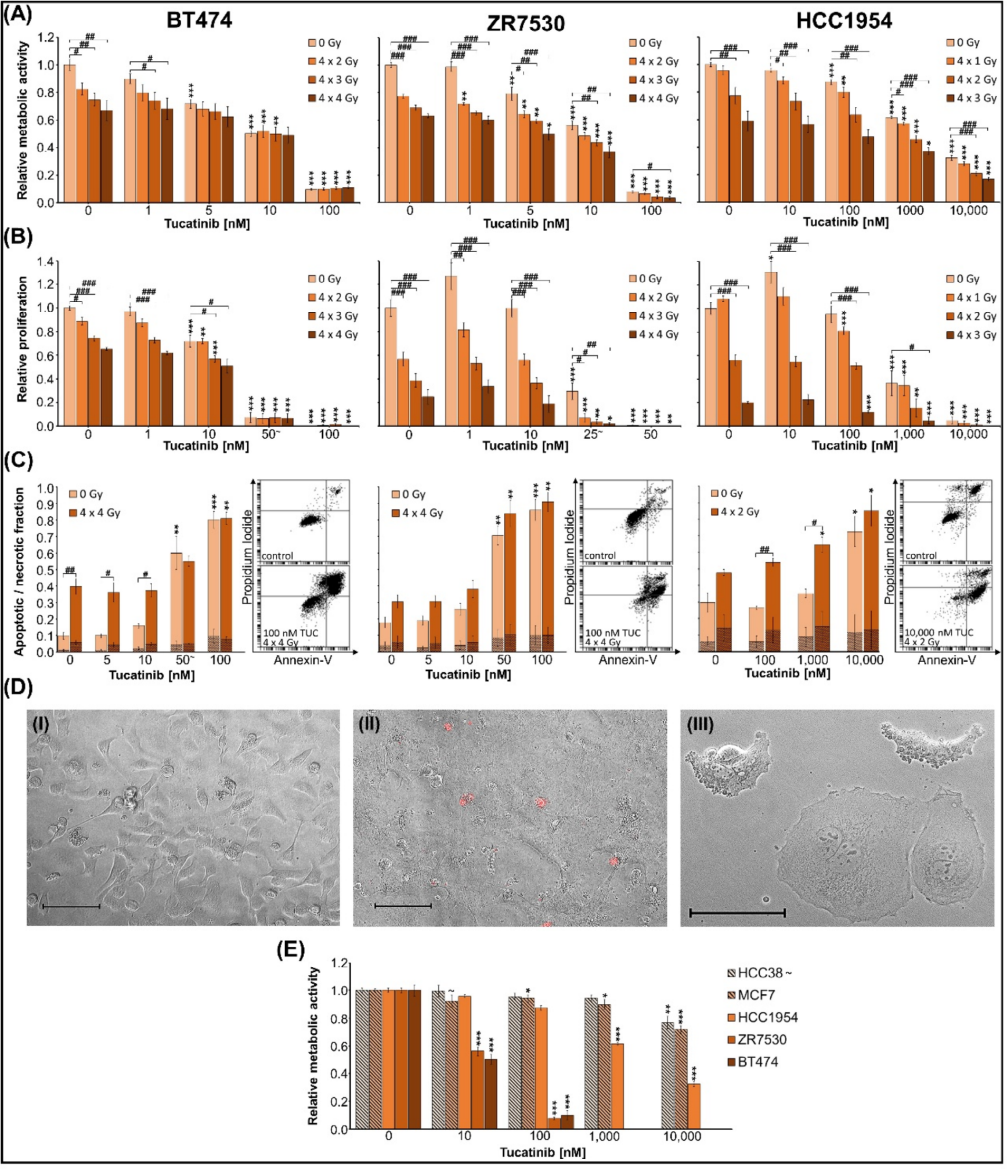
Metabolic activity, proliferation, apoptosis and necrosis of BC cell lines after combined treatment with TUC/IR. Metabolic activity, proliferation, apoptosis and necrosis of BT474, ZR7530, and HCC1954 cells 72 h after treatment with TUC and/or fractionated IR for 4 consecutive days. Data are presented as mean ± SEM of three experiments (*n* = 3) per cell line, except for ∼ (*n* = 2). Significant differences relative to the corresponding 0 nM TUC group are indicated by asterisks, between groups marked with a hash symbol. (**A**) Metabolic activity, measured by WST1 assay, and (**B**) proliferation, measured by BrdU incorporation assay, are presented relative to untreated non-irradiated control samples (= 1). Experiments were performed in duplicates. (**C**) Apoptotic (plain) and necrotic (hatched) cell fractions (1 = 100%), measured by Annexin V/PI assay, are shown together with one representative dot plot per cell line. Asterisks and hash symbols indicate significant differences in apoptotic fractions. (**D**) Phase contrast images of HCC1954 cells without (I) and 72 h after treatment (II, III) with 1 µM TUC and 8 Gy IR (red: PI; scale bar: 100 μm). (**E**) Metabolic activity, measured by WST1 assay, of HER2-negative cell lines HCC38 and MCF7 (hatched) after treatment with TUC are presented together with HER2-overexpressing cell lines (plain) relative to untreated control samples (= 1)

**Fig. 2:**
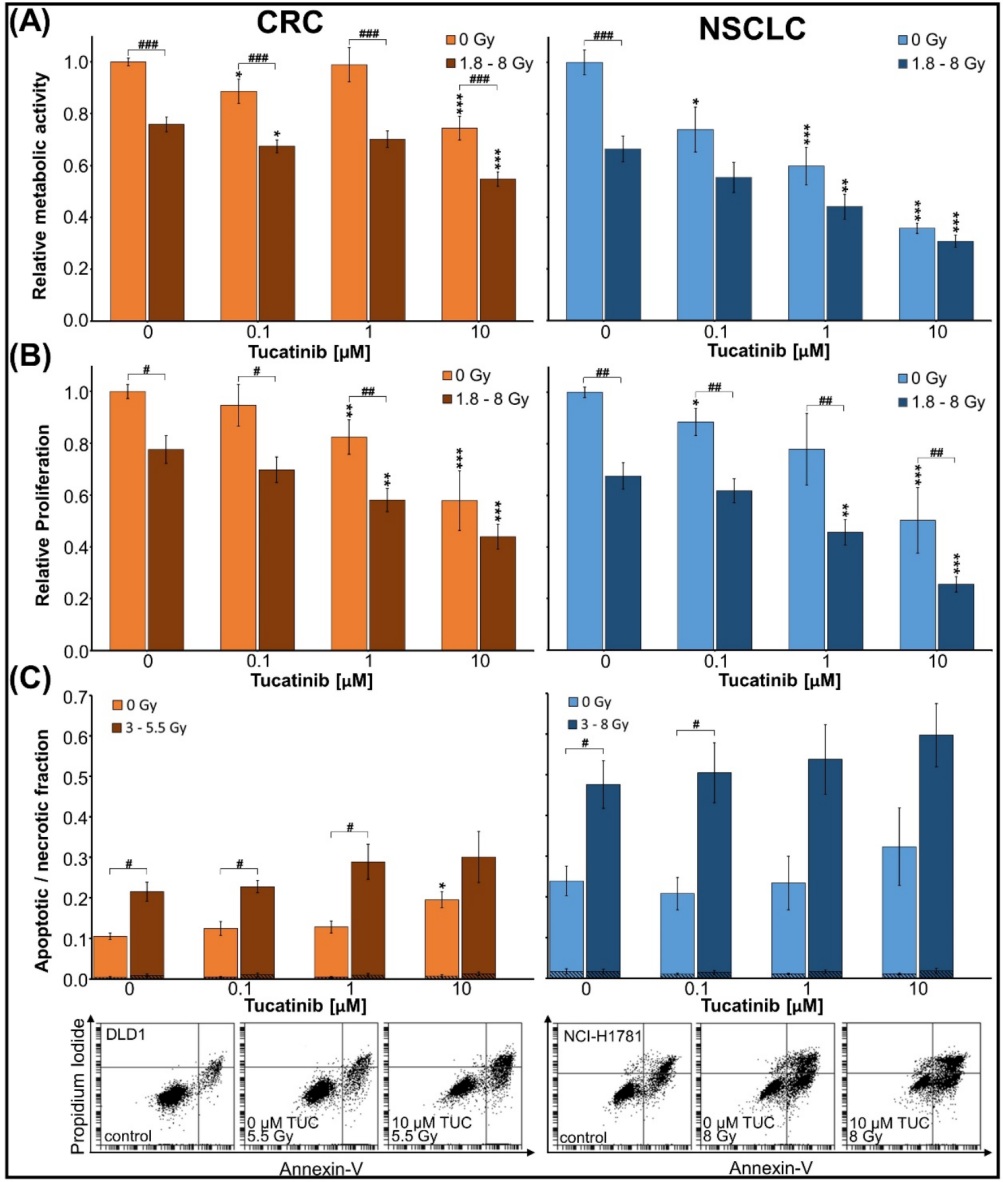
Metabolic activity, proliferation, apoptosis and necrosis of CRC and NSCLC cell lines. CRC cell lines (LS411N, DLD-1, COLO201) and NSCLC cell lines (DV90, NCI-H1781, A549) 72 h after treatment with TUC and/or single-dose IR. Data are presented as mean ± SEM of one experiment per cell line (*n* = 3). Significant differences relative to the corresponding 0 nM TUC group are indicated by asterisks between groups marked with a hash symbol. (**A**) Metabolic activity, measured by WST1 assay, and (**B**) proliferation, measured by BrdU incorporation assay, are presented relative to untreated non-irradiated control samples (= 1). Experiments were performed in duplicate (A, NCI-H1781) or triplicate (all others). (**C**) Apoptotic (plain) and necrotic (hatched) fractions, measured by Annexin V/PI assay, are shown together with one representative dot plot per entity. Asterisks and hash symbols indicate significant differences in apoptotic fractions

**Fig. 3:**
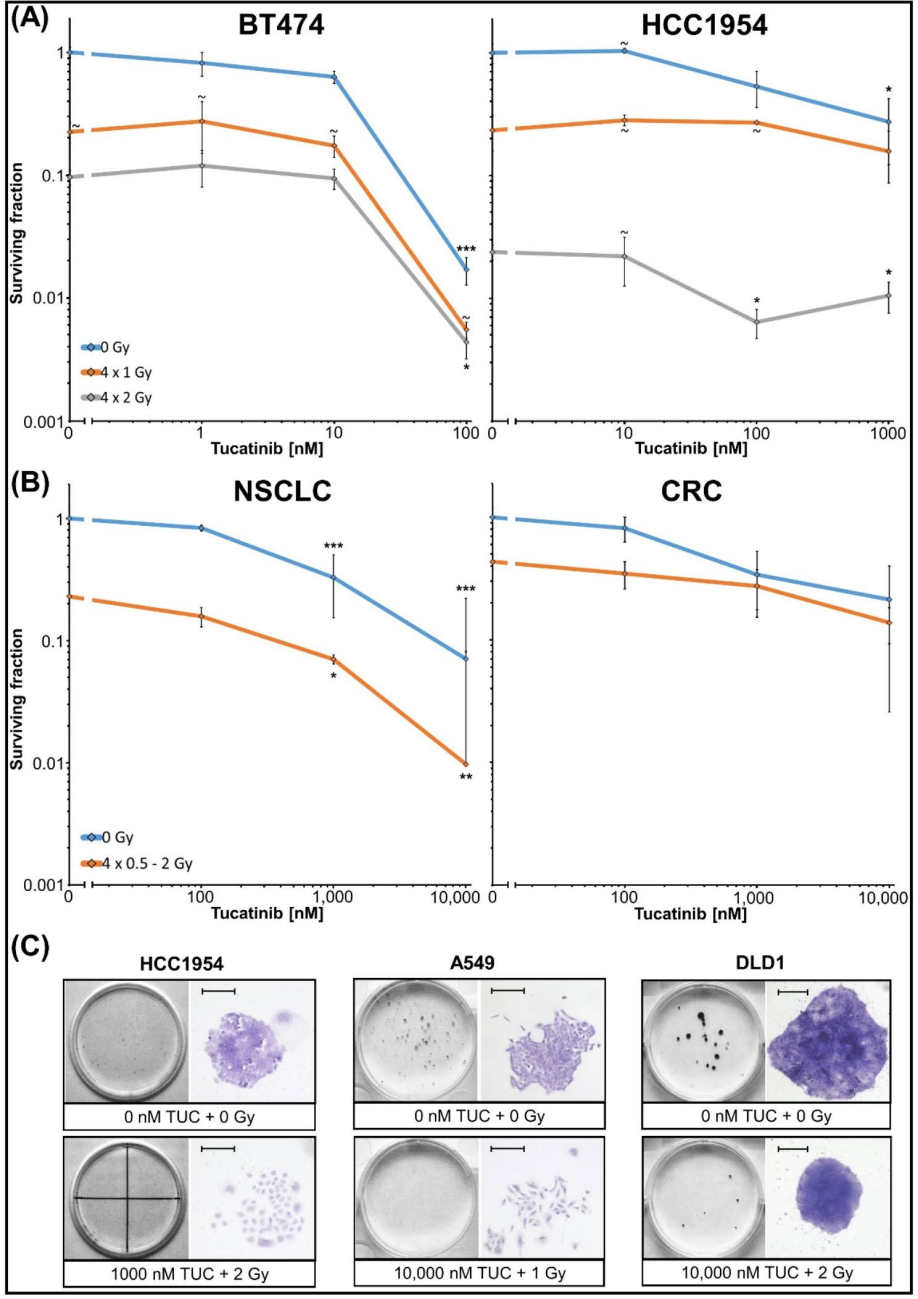
Clonogenic survival of BC, NSCLC and CRC cell lines after combined treatment with TUC/IR. Cells were treated with TUC and irradiated daily for 4 consecutive days. Significant differences to corresponding 0 Gy group are indicated by asterisks. Data are presented as mean ± SEM. (**A**) Three experiments (*n* = 3) in triplicate (BT474) or duplicate (HCC1954) are presented as mean ± SEM, except for (∼, *n* = 2). (**B**) Joint analysis was conducted for CRC (LS411N, DLD-1: triplicates, *n* = 1) and NSCLC (DV90, NCI-H1781, A549: triplicates, *n* = 1) cell lines. (**C**) Photographs of one colony together with their corresponding Petri dish/well from control as well as maximum treatment group are presented for one cell line per entity (scale bar: 300 μm)

**Fig. 4:**
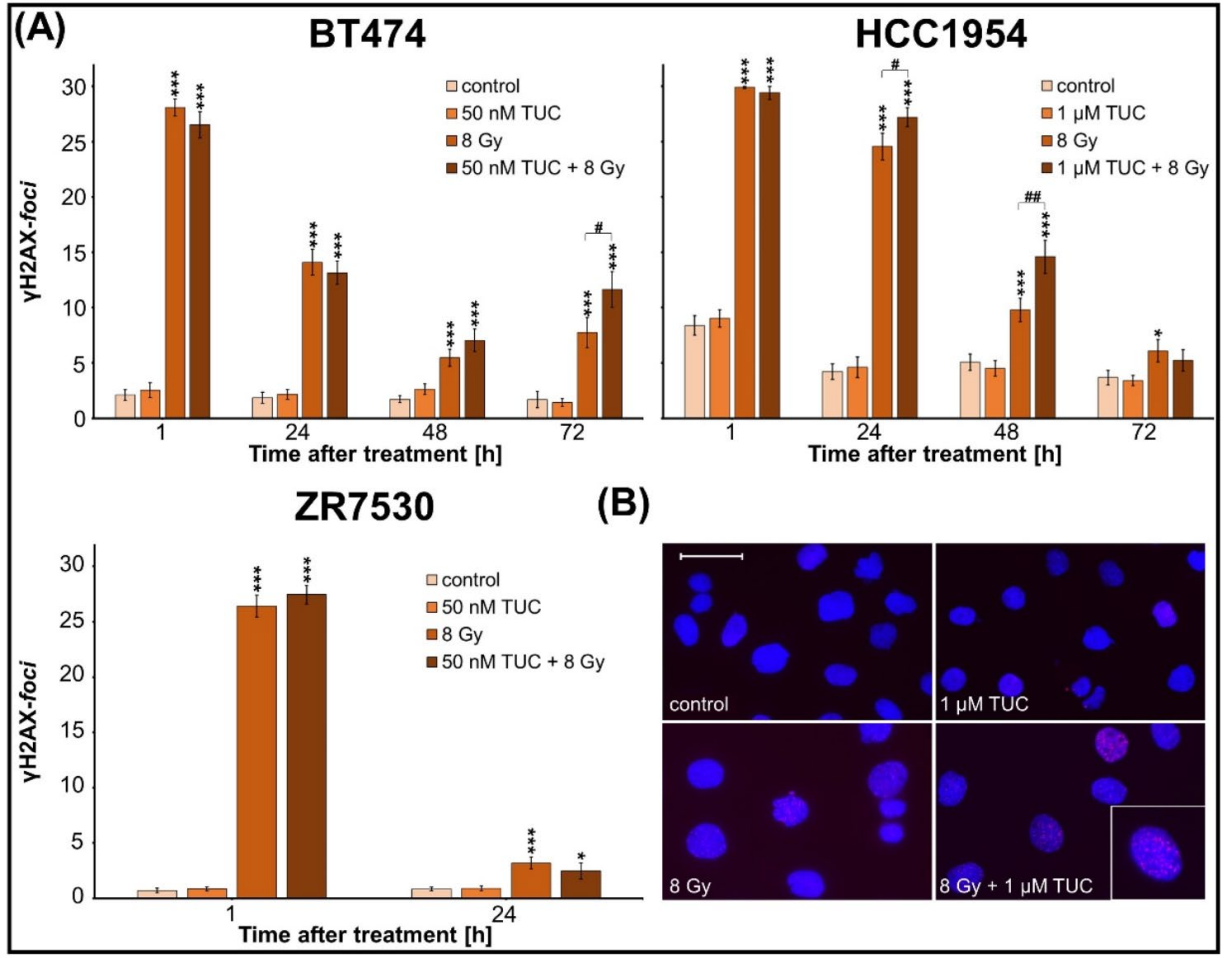
Detection of DSBs in BC cell lines after combined treatment with TUC/IR. γH2AX staining at 1, 24, 48, and 72 h after treatment of BC cell lines (BT474, HCC1954, ZR7530) with TUC and/or single-dose IR. (**A**) Number of γH2AX foci/nucleus was counted in 50 cells. Data are presented as mean value ± SEM (*n* = 1, foci of 50 cells counted). Significant differences relative to corresponding untreated control group are indicated by asterisks, between groups marked with a hash symbol. (**B**) Fluorescence images of HCC1954 24 h after TUC treatment and/or single-dose IR (blue: DAPI; red: γH2AX foci; scale bar: 50 μm; insert: 4x magnification)

**Fig. 5:**
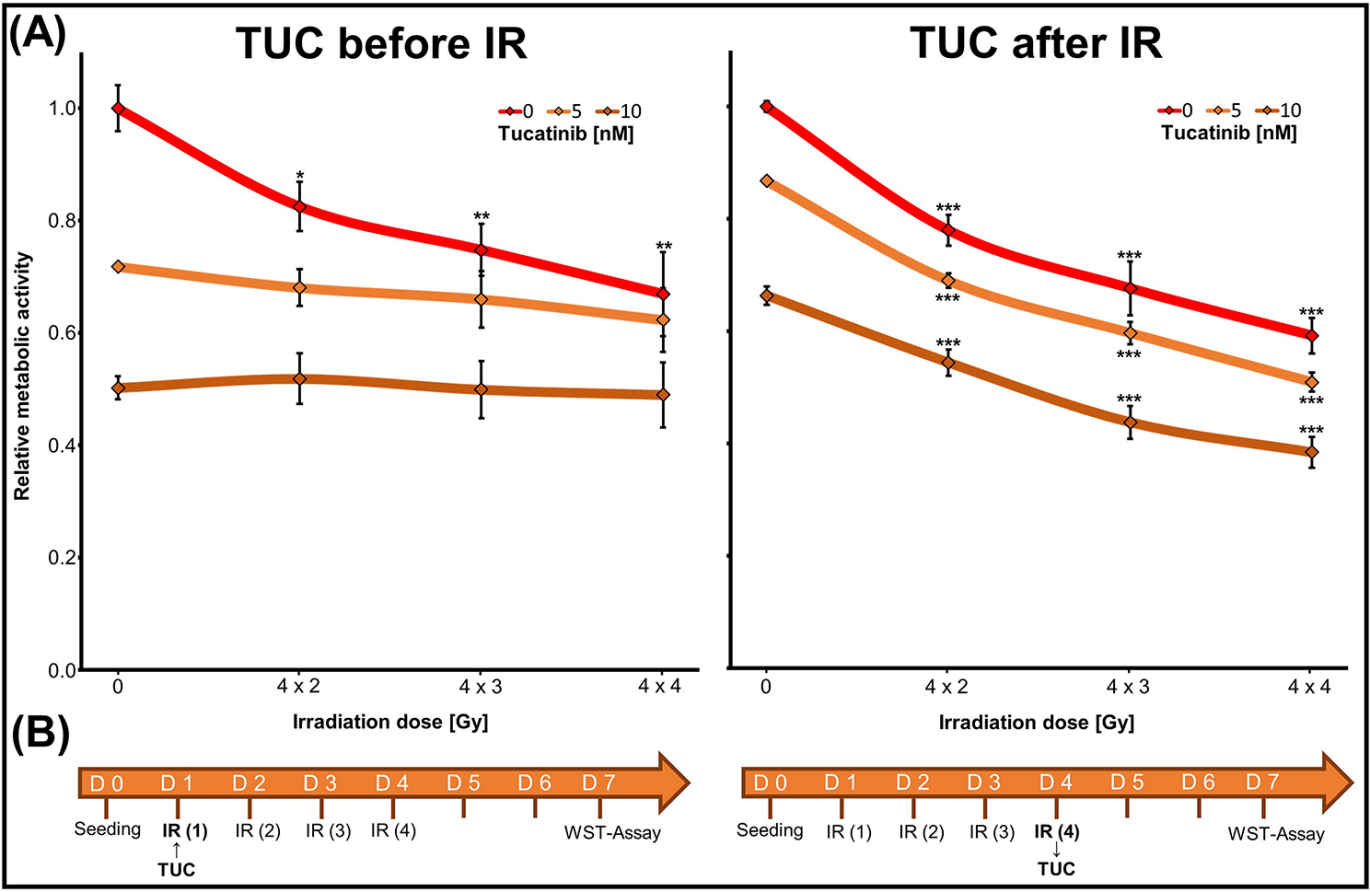
Effect of TUC/fractionated IR treatment sequence on metabolic activity of BT474 cells. (**A**) Metabolic activity was measured by WST1 assay. Data from three experiments in duplicate are presented as mean ± SEM (*n* = 3) relative to untreated control (= 1). Significant differences from the corresponding 0 Gy groups are indicated by asterisks. (**B**) Treatment schedules

**Fig. 6:**
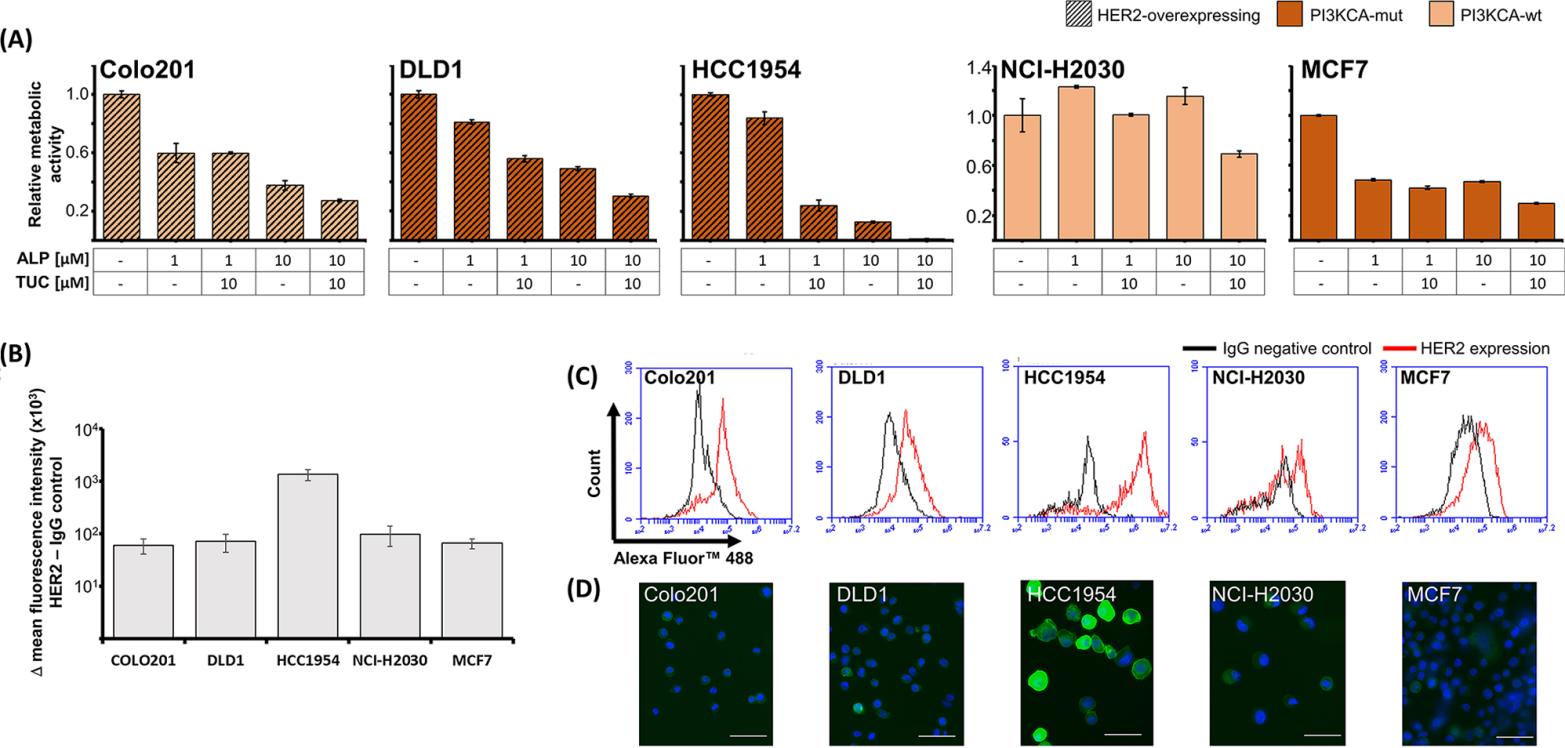
(**A**) Metabolic activity of BC, CRC and NSCLC cell lines with different PI3K and HER2 statuses after combined treatment with ALP/TUC. Metabolic activity, measured by WST1 assay, of CRC cell lines DLD1 and COLO201, NSCLC cell line NCI-H2030, and BC cell lines HCC1954 and MCF7 72 h after treatment with ALP and/or TUC. Relative values (untreated control = 1) of a single experiment performed in triplicate are presented as mean ± SEM. Cell lines are marked by shading and color as either HER2-overexpressing (hatched) or HER2-negative (plain) as well as PI3KCA-mutated (red coloring) or PI3KCA-wild type (yellow coloring). **(B)**, (**C**), (**D**) Verification of HER2 expression by Immmunofluorescence in cells used for the TUC/ALP combinatorial treatments. (**B**) Difference in mean fluorescence intensities of HER2 and IgG negative control are presented in five cell lines. Data from two independent experiments are presented as mean ± SEM. (**C**) Representative histogram overlays of HER2 staining versus IgG negative control for each cell line. (**D**) Representative photographs of HER2-(Alexa488, green) and nuclear (DAPI, blue) stained floating cells prepared for flow cytometry analysis (scale bar 50 μm)

Combinatorial effects of TUC and irradiation on metabolic activity, proliferation, and cell death were analysed in HER2-overexpressing BC cell lines (BT474, ZR7530, HCC1954), (Fig. 1). Two HER2 non-overexpressing BC cell lines (MCF7 and HCC38) were used to validate the specificity of the TUC effect (Fig. 1). To evaluate whether the results in BCs can be translated into other HER2-overexpressing entities, similar experiments have been conducted in NSCLC (DV90, NCI-H1781, A549) and CRC (LS411N, DLD1, COLO201) cell lines (Fig. 2, S1, S3). Long-term clonogenic survival was assessed in all entities after fractionated therapy (Fig. 3, S2, S4). Mechanistic insights into DNA DSB induction and repair processes are presented in Fig. 4. A possible significance of the treatment sequence was investigated exemplarily on BT474 cells (Fig. 5). To test whether a *PIK3CA* mutation in the downstream signaling pathway of HER2 is a possible target for enhancing TUC sensitivity, combinatory activity of TUC and a PI3 kinase inhibitor was examined (Fig. 6).

### Tucatinib reduces the metabolic activity in HER2-overexpressing cell lines and enhances radiation effects

#### Breast cancer

Data of BC cell lines were analyzed cell line-specific due to their individual TUC sensitivity using three independent experiments per cell line (Fig. 1A-C). TUC concentrations inhibiting the metabolic activity by 50% (IC50) ranged from 10 nM (BT474 and ZR7530) to > 1 µM (HCC1954); (*n* = 3, *p* ≤ 0.001). Fractionated IR (daily, d1-4, 1–4 Gy/fraction) significantly reduced metabolic activity in all BC cell lines by a maximum of 33 ± 8% (BT474), 37 ± 1% (ZR7530), and 41 ± 7% (HCC1954) compared to untreated control (*n* = 3; *p* ≤ 0.05). TUC treatment of irradiated BC cells significantly enhanced this effect at concentrations ≥10 nM in BT474, ≥5 nM in ZR7530, and ≥1 µM in HCC1954. Maximal combinatorial inhibition was reached at 100 nM + 4 × 2 Gy in BT474 (87±1%), 100 nM + 4 × 4 Gy in ZR7530 (95±2%) and 10 µM + 4 × 2 Gy in HCC1954 (72±3%) compared to irradiation only (*p*≤0.001). The combinatory effects of TUC and IR were also significantly higher compared to TUC alone, except for BT474 cells, where no additional effect of IR was seen after treatment with TUC≥5 nM.

To examine if the observed TUC effects are restricted to HER2-overexpressing cell lines, the metabolic activity was also measured in HER2-negative BC cell lines HCC38 (*n* = 2) and MCF7 (*n* = 3). These effects were much lower than those in HER2-overexpressing cell lines, and an IC50 could not be reached even at the highest concentration of 10 µM. At 1 µM TUC, viability was reduced by 6 ± 2% in HCC38 (n.s.) and by 10 ± 4% in MCF7 (Fig. 1E).

#### Non-small cell lung cancer and colorectal cancer

Analysis of NSCLC and CRC results was performed jointly for the cell lines of each entity (Fig. 2A; data for each cell line see Fig. S1). TUC (≥ 0.1 µM) led to a significant reduction of metabolic activity in NSCLC and CRC cell lines (*p* ≤ 0.05, *n* = 3 each). At 10 µM, metabolic activity was inhibited by 26 ± 5% (CRC) and 64 ± 2% (NSCLC); (*p* ≤ 0.001). Irradiation dose‒response experiments were done to determine the irradiation doses inhibiting the metabolic activity by 25% (ID25) for combinatory treatments. Resulting single doses ranged between 1.8 Gy (DV90/NSCLC) and 8 Gy (A549/NSCLC). The combinatorial effects of TUC and IR were significantly higher compared to IR alone. Maximal inhibition was reached at 10 µM TUC and IR (45 ± 3% in CRC; 69 ± 2% in NSCLC; *p* ≤ 0.05). In general, effects of TUC were more pronounced in NSCLC than in CRC cell lines.

### Tucatinib enhances the radiation-induced proliferation decrease

#### Breast cancer

The BC cell lines showed a significant TUC-induced reduction of proliferation at concentrations ≥10 nM (BT474), ≥25 nM (ZR7530), and ≥1 µM (HCC1954), (*n* = 3 (BT474, HCC1954), *n* = 2 (ZR7530); *p* ≤ 0.001), (Fig. 1B). However, in ZR7530 and HCC1954, a biphasic effect of TUC could be observed with an increase at the lowest tested TUC concentration (ZR7530: 1 nM, *n* = 3, n.s.; HCC1954: 10 nM, *n* = 3, *p* ≤ 0.05). Significant IR effects (doses between 4 × 1 and 4 × 4 Gy) were detected in all cell lines, with a maximum inhibition of proliferation by 35 ± 1% (BT474), 75 ± 6% (ZR7530), and 80 ± 1% (HCC1954); (*n* = 3, *p* ≤ 0.001). Combinatorial treatment resulted in additional effects of TUC to IR at concentrations of 10 nM (BT474, *n* = 3), 25 nM (ZR7530, *n* = 2), and ≥ 0.1 µM (HCC1954, *n* = 3); (*p* ≤ 0.05). At high TUC concentrations (≥ 50 nM in BT474, ZR7530 and 10 µM in HCC1954), viability was below 10%, and no additional IR effects could be detected anymore.

#### Non-small cell lung cancer and colorectal cancer

Proliferation was significantly reduced at 0.1 and 10 µM TUC in NSCLC and ≥ 1 µM in CRC (*n* = 3, *p* ≤ 0.05), with an IC50 ≈ 10 µM TUC in both entities (joint analyses Fig. 2B; data for each cell line see Fig. S1). Single-dose IR resulted in a decrease of 22 ± 5% (CRC) and 32 ± 5% (NSCLC). The combination of IR and TUC ≥ 1 µM inhibited the proliferation in both entities significantly stronger than IR alone (*n* = 3, *p* ≤ 0.01).

### Tucatinib induces apoptosis and raises radiation effects in breast cancer cell lines

#### Breast cancer

Necrotic cell fraction was higher in BC than in NSCLC and CRC, but still moderate (< 7% in control groups) with no exceeding of 16% even after maximum treatment (n. s., Fig. 1C; scatter plots see Fig. S3). Apoptotic fraction was significantly increased by TUC alone at concentrations ≥ 50 nM in BT474 and ZR7530 and at 10 µM in HCC1954 (*n* = 3, *p* ≤ 0.05). Thereby, the highest TUC concentration enhanced the number of apoptotic cells to 62 ± 5% (100 nM: BT474), 62 ± 6% (100 nM: ZR7530), and 37 ± 9% (10 µM: HCC1954) compared to control (BT474: 9 ± 2%; ZR7530: 14 ± 3%; HCC1954: 24 ± 6%). IR alone (4 × 4 Gy: BT474, ZR7530; 4 × 2 Gy: HCC1954) increased apoptosis by 25 ± 5% (BT474, *n* = 3, *p* ≤ 0.01), 10 ± 4% (ZR7530, n.s.) and 10 ± 2% (HCC1954, n.s.). Combinatorial treatment with TUC significantly enhanced these IR effects to 73 ± 4% (100 nM: BT474), 73 ± 8% (50 nM: ZR7530), and 50 ± 5% (1 µM: HCC1954); (*n* = 3, *p* ≤ 0.05). Morphological analyses of TUC- and IR-treated cells confirmed the induction of cell death by nuclear PI staining and revealed plasma membrane blebbing, a typical feature of cells undergoing apoptosis, as well as multinucleated giant cells, indicating mitotic catastrophe (Fig. 1D).

#### Non-small cell lung cancer and colorectal cancer

The fraction of necrotic cells was < 2% in all treatment groups (joint analyses Fig. 2C; data for each cell line see Fig. S1; scatter plots see Fig. S3). At 10 µM TUC, the apoptotic fraction was slightly enhanced by 9 ± 2% in CRC only (*n* = 3, *p* ≤ 0.05). IR resulted in a significant increase of apoptotic cells by 11 ± 2% in CRC and 24 ± 6% in NSCLC cell lines (*n* = 3, *p* ≤ 0.05). Combinatorial treatment with TUC and IR did not significantly increase apoptosis compared to IR alone.

### Tucatinib decreases long-term clonogenic survival and improves radiation effects

#### Breast cancer

TUC significantly reduced clonogenic survival at 100 nM (BT474; SF = 0.017 ± 0.004; *n* = 3, *p* ≤ 0.001) or 1 µM (HCC1954; SF = 0.27 ± 0.15; *n* = 3, *p* ≤ 0.05), (Fig. 3A; photographs see Fig. S4). ZR7530 could not be evaluated due to its extremely low plating efficiency (< 0.1% in the control group). IR dose-dependently diminished clonogenic survival in both cell lines. At 4 × 2 Gy, the SFs were 0.096 ± 0.01 (BT474; *n* = 2, *p* ≤ 0.001) and 0.024 ± 0.003 (HCC1954; *n* = 3, *p* ≤ 0.01). Combinatorial treatment with 4 × 2 Gy and 100 nM TUC further reduced clonogenic survival compared to IR alone, with SFs of 0.0044 ± 0.001 (BT474) and 0.011 ± 0.003 (HCC1954), (*n* = 3, *p* ≤ 0.05).

#### Non-small cell lung cancer and colorectal cancer

In CRC, clonogenic survival was assessed in LS411N and DLD1 cell lines only. COLO201 was not evaluated due to its semiadherent growth. Increasing TUC concentrations resulted in a reduction of clonogenic survival (SF = 0.34 ± 0.26 at 1 µM), (joint analyses Fig. 3B; data for each cell line see Fig. S2; photographs see Fig. S4). IR alone (between 4 × 0.5–2 Gy) decreased clonogenic survival (SF = 0.43 ± 0.05), which could be further reduced by 1 µM TUC (SF = 0.28 ± 0.1). In NSCLC cell lines, TUC (≥ 1 µM) and IR alone significantly decreased clonogenic survival (SF[TUC] = 0.33 ± 0.2 at 1 µM, SF[IR] = 0.23 ± 0.2 at 4 × 0.5–2 Gy; *n* = 3, *p* ≤ 0.001) (Fig. 3B). Combinatorial treatment showed significant additional effects at TUC ≥ 1 µM when compared to IR alone (SF = 0.07 ± 0.04 at 1 µM; *n* = 3, *p* ≤ 0.05).

### Tucatinib prolongs DNA double-strand break repair in BC cell lines

DNA double-strand break (DSB) repair kinetic alterations were investigated as possible TUC/IR-induced cell death mechanisms by γH2AX fluorescence staining in all BC cell lines (Fig. 4A, B).

No increase in the number of γH2AX foci was observed after TUC treatment in any of the three cell lines, indicating that TUC itself did not induce DSBs. In contrast, the number of initial γH2AX foci was significantly enhanced in all cell lines 1 h (*p* ≤ 0.05) after IR and to a similar degree after combined IR/TUC treatment, without showing an additional TUC effect. The number of residual IR-induced foci decreased in a time-dependent manner (24–72 h), with the highest repair rate found in ZR7530 cells. Compared to IR alone, the combined IR/TUC treatment significantly enhanced the residual foci number in BT474 at 72 h (7.8 vs. 11.6 foci; *p* ≤ 0.05) and in HCC1954 cells at 24 h (24.5 vs. 27.2 foci; *p* ≤ 0.05) and 48 h (9.8 vs. 14.6 foci; *p* ≤ 0.01).

### Treatment sequence of tucatinib administration influences the radiation sensitivity of the BC cell line BT474

To examine a possible impact of the treatment sequence on the combinatorial anti-tumor activity of TUC and IR, we compared two treatment schedules exemplarily in BT474 cells (Fig. 5). When TUC (5 or 10 nM) was applied before IR (4 × 2, 3, or 4 Gy), no additional IR effect was found. In contrast, when TUC was added after IR, we observed a significant additional reduction of metabolic activity at all IR doses. At 4 × 4 Gy, the TUC effect was reduced by 36 ± 1% (5 nM) and 28 ± 3% (10 nM) compared to treatment with TUC alone (*n* = 3, *p* ≤ 0.01) (Fig. 5A, B).

### Alpelisib enhances tucatinib effects in the low-sensitive, *PI3KCA*-mutated BC cell line HCC1954

In our experiments, we observed a lower sensitivity to TUC in the HER2-overexpressing BC cell line HCC1954 compared to the HER2-overexpressing BC cell lines BT474 and ZR7530. To evaluate whether the *PIK3CA* gene mutation in the downstream signaling pathway of HER2 in HCC1954 cells is responsible for this partial TUC resistance, we applied a PI3KCA inhibitor (alpelisib, ALP) and analyzed the metabolic activity of three *PI3KCA*-mutated and two *PI3KCA*-wildtype cell lines (see Table 2) after combination treatment with TUC and ALP (Fig. 6A).

We observed a dose-dependent reduction of metabolic activity by ALP in all *PI3KCA*-mutated cell lines (HCC1954, DLD1, MCF7) but also in the *PI3KCA*-wildtype COLO201 cell line. Only the *PI3KCA*-wildtype and non-HER2-overexpressing NCI-H2030 cell line was not affected by ALP as a single agent. TUC at maximum concentration (10 µM) diminished the metabolic activity most strongly in the HER2-overexpressing cell lines (HCC1954, DLD1, COLO201) and to a lesser extent in (HER2 low-expressing) MCF7 cells. NCI-H2030 cell line, possessing neither *PI3KCA* mutation nor HER2 overexpression, proved to be the most resistant cell line.

The combination of 10 µM TUC and 10 µM ALP (the highest concentrations employed) differentially reduced the metabolic activity in all tested cell lines. According to their *PI3KCA* mutation or HER2 expression status, lowest effects were achieved in the NCI-H2030 and highest response in the HCC1954 cell line.

**Table 2:** *PIK3CA* and HER2 status in five cancer cell lines

Cell line (entity)	PI3KCA status	HER2 status
HCC1954 (*BC*)	mut	HER2 overexpression
MCF7 *(BC)*	mut	no HER2 overexpression
DLD1 *(CRC)*	mut	HER2 overexpression
COLO201 *(CRC)*	wt	HER2 overexpression
NCI-H2030 *(NSCLC)*	wt	no HER2 overexpression

*PI3KCA* mutation status of indicated cell lines was evaluated via depmap.org [35, 37–40] and is characterized either by wt = wildtype or mut = mutated. HER2 expression status is based on upper mentioned sources.

Re-evaluation of HER2 expression levels in these five cell lines by flow cytometry mainly confirmed the literature based assumptions. The HER2-overexpressing HCC1954 cells showed a 20-fold higher HER2 fluorescence intensity (mean) compared to the non-overexpressing MCF7 breast cancer cell line. The HER2-overexpressing colon cancer cell lines DLD1 and COLO201 but also the non-overexpressing NSCLC cell line NCI H2030 showed between 15 and 30% of HER2-positive cells albeit with lower expression levels, similar to MCF7 cell line (Fig. 6B, C).

Fluorescence microscopy confirmed these results indicating mainly membrane localization of the HER2 staining (Fig. 6D), [38, 40–43].

## Discussion

In the clinical setting, for HER2-positive BC brain metastasis survival times between 10 and 25 months are reported after radiotherapy, involving whole brain (WBRT) or stereotactic radiotherapy (SRT) [44–46]. However, local recurrences are frequent. After radiotherapy, local failure rate in HER2-positive BC appears higher than in HER2-negative BC patients, implying relevant radioresistance and a need for additional “consolidating” systemic treatment.

Consequently, we investigated here in vitro effects of a combinatorial treatment with IR and the HER2 tyrosine kinase inhibitor TUC in HER2-overexpressing human cancer cell lines. TUC is currently approved for the treatment of HER2-positive, metastatic BC and was approved by the FDA for the treatment of HER2-positive, metastatic CRC in 2023 (see above), whereas in advanced NSCLC, an unmet need remains for HER2-targeted therapy. Data investigating the possible benefit of combination therapy with IR are missing so far and therefore have been evaluated here.

The efficacy of anti-HER2 monotherapy in BC has been well described, where high HER2 expression is an important oncogenic driver [47]. In other tumor entities HER2-targeted therapies have shown mostly disappointing results [48, 49], indicating the presence of additional/other driver mutations or heterogeneous HER2 receptor expression. Previous experiments demonstrating disproportionately higher HER2 expression in the BC cell line BT474 than in the CRC cell line DLD1 and NSCLC cell line A549 [30] might partially explain the entity-specific TUC responses in our experiments and are in line with recent in vitro studies [13, 50]. Moreover, we demonstrated reduced cytotoxic activity of TUC in HER2-low-expressing compared to HER2-overexpressing BC cell lines (Fig. 1). These findings support the high specificity of TUC [13] and indicate that the potency of this TKI at physiological concentrations is strictly related to the expression of the HER2 receptor, making its application in HER2-negative cancer ineligible.

In accordance with its predicted inhibition of HER2 signaling pathways involving MAPK and PIK3/AKT activation [4, 51], TUC significantly reduced cell proliferation and induced cell death in all investigated HER2-overexpressing cell lines. Thereby, a biphasic TUC effect might be indicated by slight initial proliferation increases in 2/3 of HER2-overexpressing BC cell lines at low TUC concentrations. This could be related to proliferative activation and upregulation of other EGF receptor types at subtherapeutic TUC concentrations. So, others have shown that inhibition of one EGF receptor type can upregulate other EGF receptor family members [52]. Cell death was efficiently induced in BC by TUC, mainly through apoptotic pathways and only marginally by necrosis. In NSCLC and CRC, the number of apoptotic cells was much lower despite the higher TUC concentrations than in BC. Therefore, the observed changes in metabolic activity in these entities might be rather due to TUC-induced proliferation inhibition than to TUC-induced cell death. Nevertheless, a TUC concentration of 100 nM significantly reduced the metabolic activity of all examined HER2-overexpressing cell lines.

For long-term clonogenic cell death induction, again, higher TUC concentrations (≥ 1 µM) had to be applied in NSCLC and CLC compared to BC cell lines. In TUC standard-dosed patients (2 × 300 mg/d), physiological concentrations > 1 µM TUC are unlikely to be achieved. Pharmacological studies reported a maximum plasma concentration between 532 and 790 ng/ml (1.21–1.64 µM) [53, 54] and a modeled mean liquor concentration of 5.37 ng/ml (11 nM) [55]. The effective concentration required for our in vitro experiments suggested that TUC might be active as monotherapy in BC but rather not in CRC or NSCLC. To investigate combined effects of TUC and radiotherapy, IR dose-finding experiments were conducted using viability tests. Much higher IR doses in BC than in NSCLC and CRC cell lines were needed; for BC experiments, also a fractionated setting was used. These findings support the reported association of HER2 alterations with radioresistance [17, 31] and are going along with the lower HER2 receptor expression in NSCLC and CRC cell lines (see above).

The addition of TUC increased the anti-tumor effects on metabolic activity, proliferation, and clonogenic survival, particularly in HER2-overexpressing BC cells, compared to irradiation alone; however, in contrast to the data shown for trastuzumab or lapatinib, no radiosensitizing effect was found [17, 18]. Drug response/gene profiling analyses have demonstrated distinct TKI-specific activities due to their differential receptor binding, which might account for such differences [50].

Nevertheless, the results indicate that combined treatment with radiotherapy and TUC might be superior to monotherapy. The data from the HER2CLIMB trial (TUC in combination with trastuzumab and capecitabine, which included 291 patients with brain metastases; showing a prolongation of the median PFS in the CNS from 4.2 to 9.9 months) point out that TUC is highly active in brain metastases and that a relevant proportion of patients do not or only shortly benefit from TUC treatment [11, 56]. Together with the limited therapeutic activity of standard radiation therapy (above), our data support its high potential for clinical application.

Combinatory effects of TUC and IR were again less pronounced in NSCLC and CRC cells, which is in concordance with clinical studies examining different HER2-targeted therapies in these entities [25, 29]. Thereby it is currently not fully understood to what extent distinct HER2 alterations (e.g., overexpression, amplification, point mutations) can predict the clinical outcome in NSCLC and CRC [25, 57].

To gain more insight into the mechanism of the anti-tumor activity of combined TUC/IR treatment, we investigated its effect on double-strand brake induction, a key mechanism in the antineoplastic activity of IR. More importantly, upregulated DNA repair has been suggested to be a radioresistance mechanism in HER2-overexpressing tumors, and in vitro studies using the dual EGFR/HER2 inhibitor lapatinib showed about 100% more residual DNA damage foci by combined treatment compared to IR alone in HER2-overexpressing SKBR3 cells (BC) [18]. Similarly, we detected up to 50% more residual DSB foci after combinatorial TUC and IR treatment in BT474 and HCC1954 BC cells than after IR alone, indicating interference of TUC in DSB repair as a potential mechanism of action. This is in line with the reported downregulation of CDK12, which affects the DNA damage response [58] by TKIs, including TUC [50]. Nevertheless, no significant radiosensitization could be found in clonogenic assays, suggesting that this mechanism is of subordinate relevance in TUC/IR treatment.

With respect to the treatment sequence, pretreatment with high TUC concentrations prior to the first IR fraction resulted in reduced additional radiation effects, especially in the BT474 cell line. We hypothesized that the anti-proliferative activity of TUC might diminish radiosensitivity [59]. Indeed, significant IR effects were observed only when TUC was applied after the last IR fraction, supporting our hypothesis. Others reported no effect of treatment sequence using trastuzumab but applied a non-fractionated IR and a fractionated drug schedule [60]. From our data, it can be postulated that a sequential treatment with IR prior to TUC might have the greatest effect on preventing resistance to either of these treatments.

When HCC1954 BC cells showed less sensitivity to TUC than the other examined BC cell lines, we hypothesized that this might be due to activating mechanisms downstream of the HER2 signaling pathway. In fact, HCC1954 harbors a gain-of-function mutation in the α-catalytic subunit of phosphatidylinositol-3 kinase (PI3KCA) [61], leading to a resistance against HER2-targeting antibody trastuzumab. PI3K is an enzyme downstream of HER2 that plays a role in mediating cell growth, proliferation, and overall survival [4]. Accordingly, the application of a PI3K inhibitor increased trastuzumab sensitivity [62]. Based on these findings, we combined TUC with the PI3KCA inhibitor alpelisib (ALP) and found that the restored TUC response in HCC1954 cells supports a causal role of the *PI3KCA* mutation in TUC resistance, which was confirmed in the *PI3KCA*-mutated DLD-1 CRC cell line. Currently, patients with *PIK3CA*-mutant, HER2-positive, metastatic BC are being recruited for a clinical trial with ALP and TUC [63]. Prospective studies might benefit from our first in vitro results in which IR was introduced into this setting. Given the facts that approximately 20–50% of all BCs harbor a *PIK3CA* mutation and that HER2-positive BCs are most commonly positive for this mutation (23%), this combination seems to be of high clinical relevance [64].

Surprisingly, the viability of *PI3KCA*-wildtype COLO201 cells was also reduced by ALP, which might be related to its elevated EGFR levels [33], as PI3K-activated pathways are an influential part of the EGFR signaling network [4]. Because COLO201 is also *BRAF*-mutated, another possible explanation may be the inhibition of important BRAF-downstream pathways. So, it has been previously shown that this can be achieved by targeting PI3K [65], broadening its potential clinical application.

The inhibition of NCI-H2030 cells (negative control, [37, 38]) by simultaneous treatment with TUC and ALP (10 µM; corresponding to the potentially achievable concentration in humans [66, 67]) indicates nonspecific toxic effects at high drug concentrations, possibly through the inhibition of physiological PI3K and EGFR levels.

Moreover, re-evaluation of literature-based HER2 expression status demonstrated low level HER-2 expression in 28% of NCI H2030 cells.

In light of these results, the high TUC concentrations needed to achieve significant anti-neoplastic activity in CRC and NSCLC cells may not only be the result of low HER2 expression levels but also indicate an important role of additional driver and resistance mechanisms in these entities. In concordance, two of our three NSCLC cell lines harbor *KRAS* mutations [68–70], and all CRC cell lines exhibit WNT and/or MAPK pathway alterations [71], which reflects the clinical situation [72].

Further studies may examine whether targeting HER2 aberrations with antibody‒drug conjugates to compensate for the low expression levels and/or simultaneous inhibition of additional driver events, together with IR, might enhance the therapeutic efficacy of HER2 inhibitors in these entities and preventing regrowth of resistant cells.

## Conclusion

In this in vitro study, combinatorial treatment of HER2-overexpressing BC with IR and TUC was more effective than corresponding single treatments, warranting further in vivo investigations. Dual treatment might be especially important for brain-metastasized BC patients, for whom new therapeutic strategies improving patient outcomes are urgently needed. TUC combined with IR also demonstrated efficacy in HER2-overexpressing NSCLC and CRC cell lines, albeit at higher concentrations only. The treatment sequence might also be relevant and should be validated in vivo evaluation. Combined targeting of HER2 downstream aberrations such as *PI3K* mutations could aid in overcoming resistance to TUC and possibly broaden the responsive entities.

### Electronic supplementary material

Below is the link to the electronic supplementary material.

Supplementary Material 1

Supplementary Material 2

Supplementary Material 3

Supplementary Material 4

## Data Availability

The datasets used and/or analyzed during the current study are available from the corresponding author upon reasonable request.

## Abbreviations

TUC: Tucatinib
IR: Irradiation
BC: Breast Cancer
CRC: Colorectal Cancer
NSCLC: Non-Small Cell Lung Cancer
ALP: Alpelisib
IC/ID: Inhibitory Concentration/Inhibitory Dose
DSB: Double Strand Breaks
