# Supplementary figures and images for: Enhanced anti-tumor effects by combination of tucatinib and radiation in HER2-overexpressing human cancer cell lines
Source: Cancer Cell Int. 2024 Aug 6;24:277. doi: 10.1186/s12935-024-03458-3 (PMC11302197; doi:10.1186/s12935-024-03458-3)

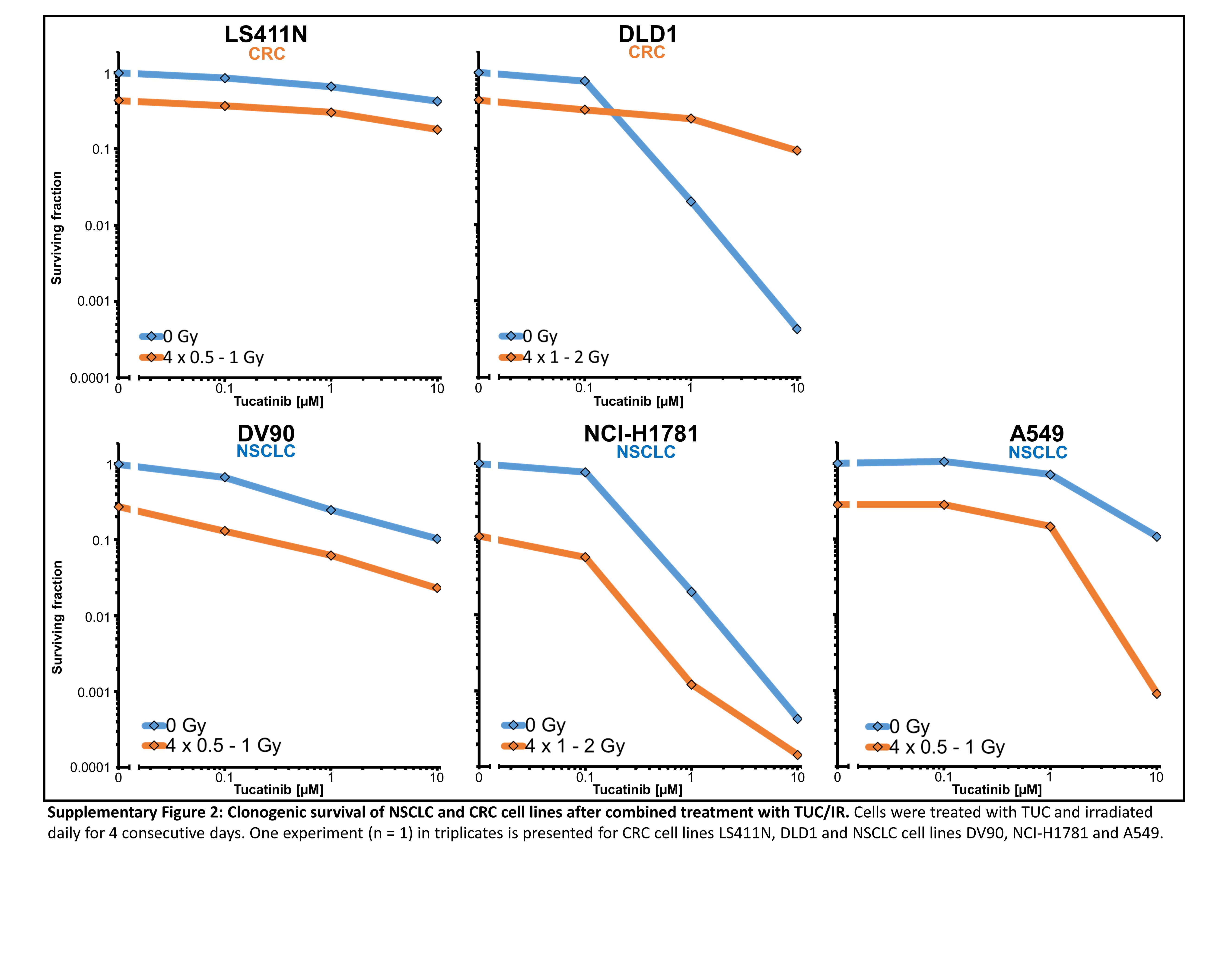

Supplement: Supplementary file 1 — Supplementary Material 1 [file 12935_2024_3458_MOESM1_ESM.jpg]

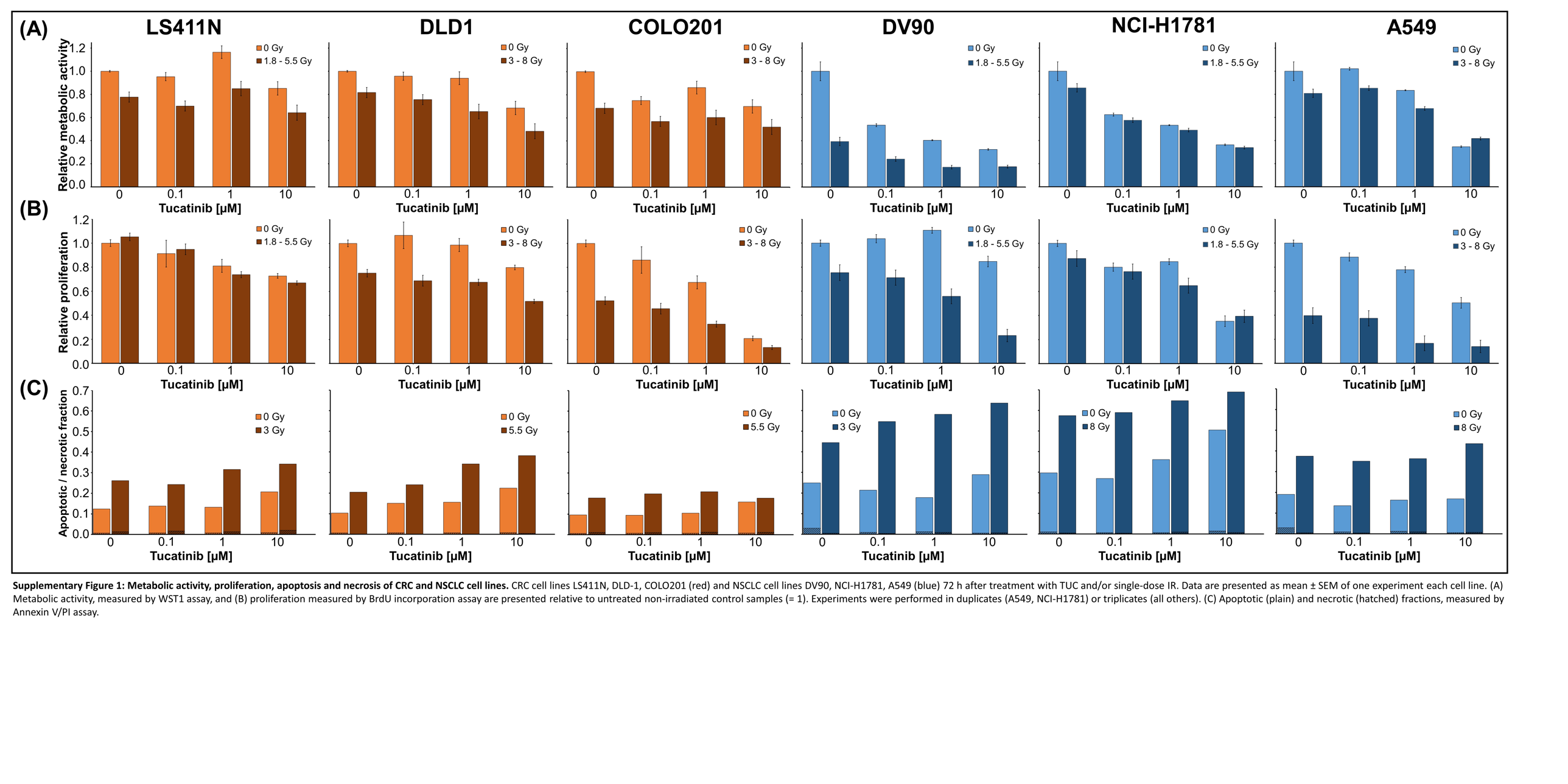

Supplement: Supplementary file 2 — Supplementary Material 2 [file 12935_2024_3458_MOESM2_ESM.jpg]

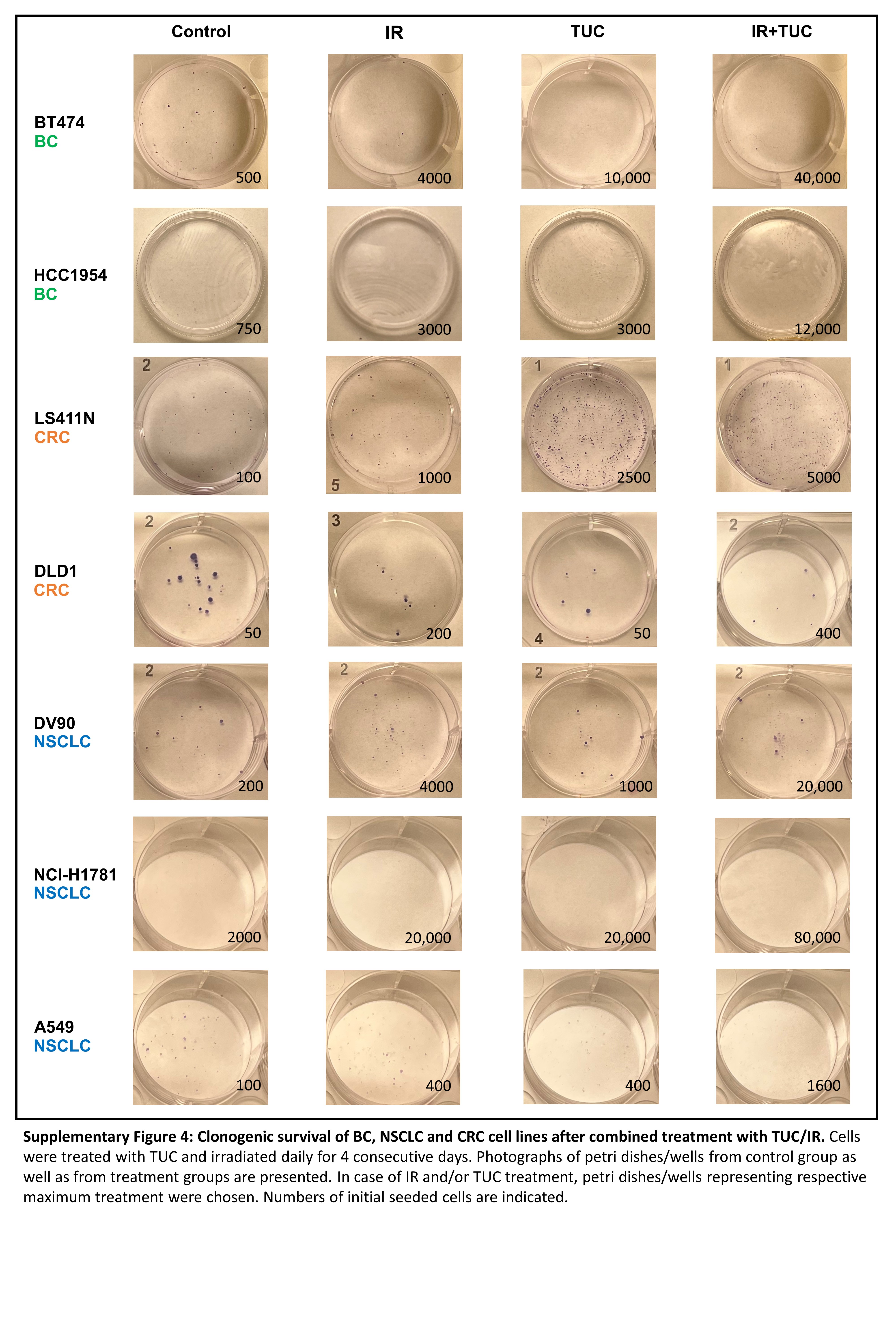

Supplement: Supplementary file 3 — Supplementary Material 3 [file 12935_2024_3458_MOESM3_ESM.jpg]

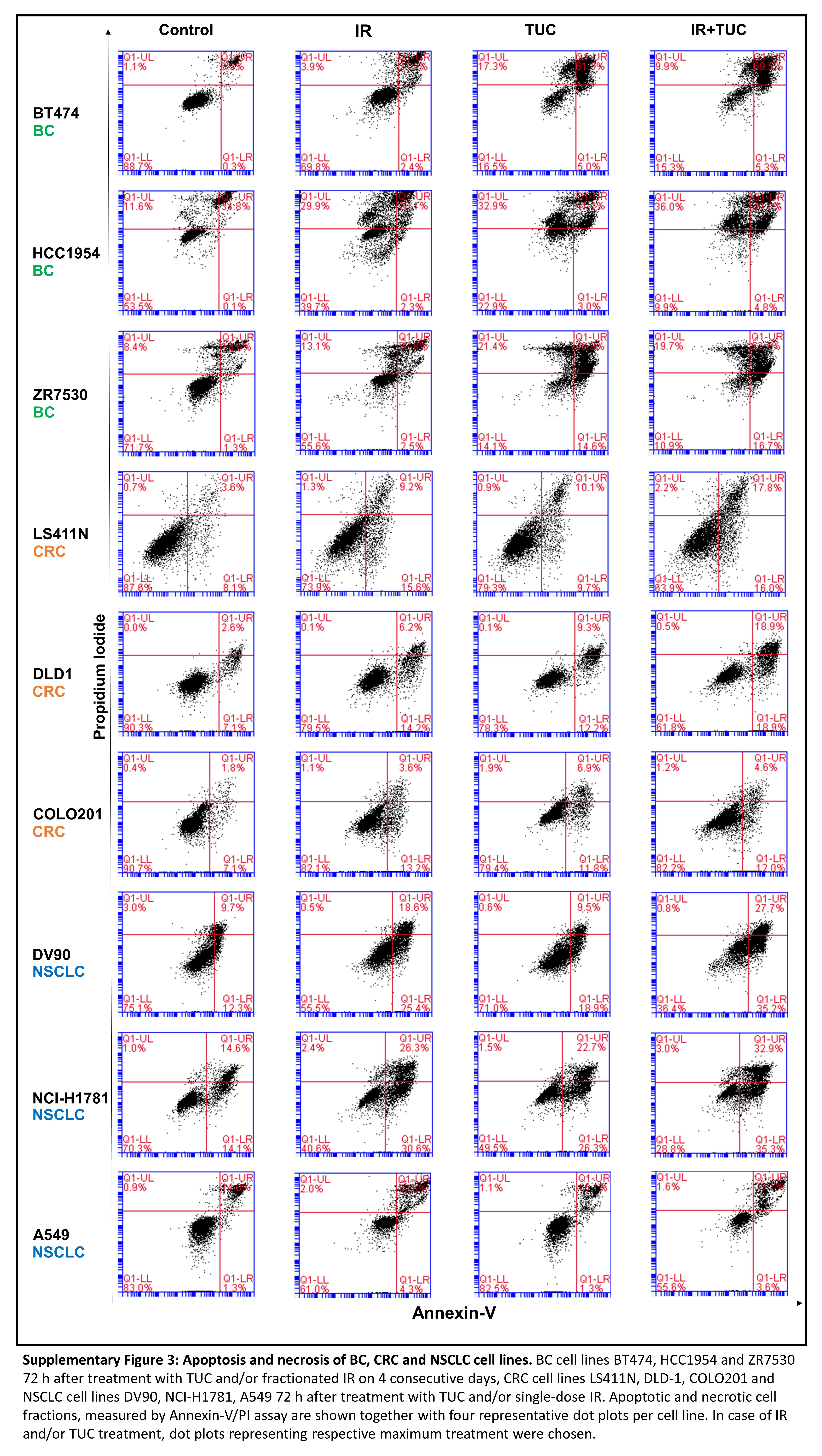

Supplement: Supplementary file 4 — Supplementary Material 4 [file 12935_2024_3458_MOESM4_ESM.jpg]
